# Carbon dioxide emission-intensity in climate projections: Comparing the observational record to socio-economic scenarios

**DOI:** 10.1016/j.energy.2017.06.119

**Published:** 2017-09-15

**Authors:** Felix Pretis, Max Roser

**Affiliations:** a Department of Economics, University of Oxford, United Kingdom; b INET at the Oxford Martin School, University of Oxford, United Kingdom; c Programme of Empirical Research into Innovation and Changing Living Conditions at the Oxford Martin School, University of Oxford, United Kingdom

**Keywords:** Energy production, Emission intensity, Climate change, Scenarios, IPCC, Prediction

## Abstract

The wide spread of projected temperature changes in climate projections does not predominately originate from uncertainty across climate models; instead it is the broad range of different global socio-economic scenarios and the implied energy production that results in high uncertainty about future climate change. It is therefore important to assess the observational tracking of these scenarios. Here we compare these socio-economic scenarios created in both 1992 and 2000 against the recent observational record to investigate the coupling of economic growth and fossil-fuel CO_2_ emissions. We find that global emission intensity (fossil fuel CO_2_ emissions per GDP) rose in the first part of the 21st century despite all major climate projections foreseeing a decline. Proposing a method to disaggregate differences between scenarios and observations in global growth rates to country-by-country contributions, we find that the relative discrepancy was driven by unanticipated GDP growth in Asia and Eastern Europe, in particular in Russia and China. The growth of emission intensity over the 2000s highlights the relevance of unforeseen local shifts in projections on a global scale.

## Highlights

• The wide range of socio-economic scenarios in climate projections results in high uncertainty about climate change.
• We compare socio-economic scenario projections to observations over 1990–2010.
• Global CO_2_ emission intensity increased despite all major scenarios projecting a decline.
• Under-projection of emission intensity raises concerns about achieving emission targets.

## 1 Introduction

The wide spread of projected temperature changes in climate projections does not predominately originate from uncertainty across climate models; instead it is the broad range of different global socio-economic scenarios and the implied energy production that results in high uncertainty about future climate change. While the physical-science basis of models used in Intergovernmental Panel on Climate Change (IPCC 1990–2013) [8] reports is very much the focus of the debate in climate research [23], the underlying socio-economic scenarios that determine emissions of greenhouse gases have received comparably less attention. Observations over two decades are now available against which the initial sets of socio-economic scenarios underlying the IPCC reports can be assessed to study the observational tracking. Here we compare these socio-economic scenarios created in both 1992 (IS92 – see Refs. [12], [20]) and 2000 (SRES – see Ref. [17]) against the recent observational record to investigate the coupling of economic growth and fossil-fuel carbon dioxide (CO_2_) emissions. We find that global emission intensity (fossil fuel CO_2_ emissions per real gross domestic product - GDP) rose in the first part of the 21st century, despite all major climate projections foreseeing a decline. Studying the differences between projections and observations we find that the relative discrepancy was driven by unanticipated GDP growth in wider Asia, particularly in Russia and China. The growth of emission intensity over the 2000s highlights the relevance of unforeseen local shifts in projections on a global scale.

We make three main contributions to the existing literature. First, we provide an assessment of socio-economic scenarios in terms of their growth rates over a long time-span matching the intervals of the IPCC scenarios. The assessment is particularly relevant to investigate any suggested de-coupling of economic growth from fossil-fuel CO_2_ emissions. Second, we provide a method to decompose aggregate differences in growth rates into individual contributions when down-scaling is necessary due to a coarser resolution of the projected values relative to observational data. This can be used for future scenarios on a global level to assess whether particular countries (or regions) have led to systematic deviations from the projected paths. Our analysis highlights that unforeseen shifts in single countries can contribute substantially to global differences and our decomposition allows this contribution to be quantified directly. Third, based on our conclusions we provide suggestions for the development of future scenarios.

Four sets of scenarios have been used in the IPCC reports: the first used the SA90 scenarios, the second used the IS92 projections, the third and fourth used the Special Report on Emission Scenarios (SRES), while the fifth relied on the Representative Concentration Pathways (RCPs)^2^. We assess all main socio-economic indicators in the IS92 and SRES: GDP, population, and fossil fuel CO_2_ emissions, all of which are major components in the Kaya identity [11]. We focus on emission intensity as it is a crucial measure for the environmental impact of energy production and economic growth, and plays a crucial role in projected future warming [1]. Emission intensity combines two projected socio-economic series, GDP and fossil-fuel CO_2_ emissions^3^. Additional results for all other socio-economic series are reported in the supplementary material.

**Fig. 1:**
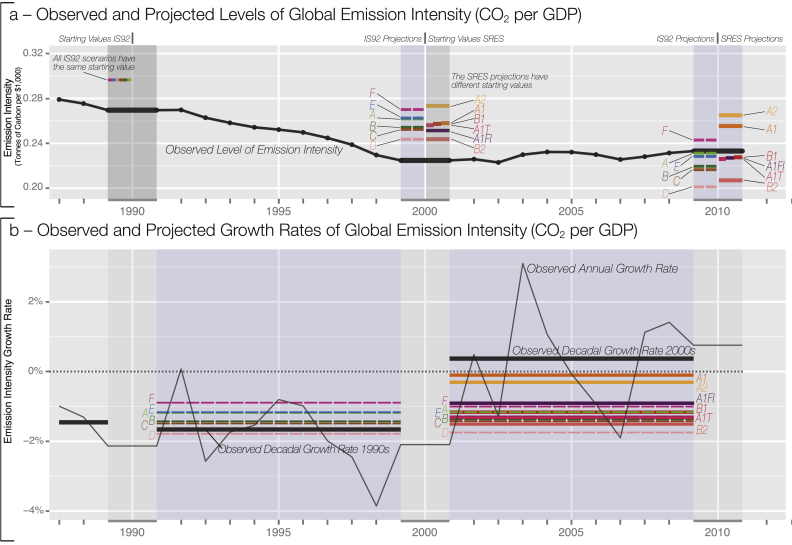
Observed (black) and projected (colour) global emission intensity in levels (a) and growth rates (b). Panel a graphs global observed emission intensity together with decadal IS92 and main SRES marker projections. Panel b shows observed annual growth rates together with observed decadal growth rates over both decades. Observed decadal growth rates exceed all main scenario projections over the 2000s. (For interpretation of the references to colour in this figure legend, the reader is referred to the web version of this article.)

There are six socio-economic paths in the 1992 scenarios (named A-F). The later SRES projections are made up of multiple individual scenarios falling within four broad groups: A1 (rapid growth and convergence, where A1FI denotes fossil fuel intensive while A1T concentrates on non-fossil fuels), A2 (heterogeneous development), B1 (convergence and ecologically friendly), and B2 (heterogeneous and ecologically friendly). As the use of sub-group averages is not appropriate [13], we instead focus on the six main ‘illustrative’ SRES marker scenarios^4^ together with the six IS92 scenarios. We also report the results for the full set of all SRES scenarios. Emission scenarios used in the early IPCC climate models are reported at a decadal interval (1990, 2000, 2010) precluding us from assessing the sensitivity of projections to the choice of time interval. Different SRES scenarios were allowed varying initial values at the start of the scenario projections (see Fig. 1 panel *a,* and Supplementary Figs. S1–S2). This results in small differences for global population (up to 0.38%) but in large differences in world GDP and CO_2_ emissions – up to 4.3% between scenarios A2 and B1. This variation in starting values stems from uncertainty around observed GDP and socio-economic variables and complicates any study of the accuracy in levels. Discrepancies in initial values make it necessary to focus on growth rates. Since scenarios cannot be expected to capture short-term year-on-year fluctuations, a comparison on a decadal scale is appropriate. Crucially, for the future development of scenarios it is important to ensure consistent initial values across all projections despite intrinsic uncertainties around observations. This approach has been embraced by the RCP scenarios in the latest IPCC assessment.

Unlike temperature forecasts derived from climate model ensembles that are conditioned on scenarios, the socio-economic scenarios themselves are not probabilistic projections, but are meant to describe possible future pathways. Nevertheless, it is interesting to see whether any of the possible paths have been realised, and to provide guidance for conditioning when assessing the accuracy of climate forecasts. Uncertainties on observed CO_2_ emissions and GDP are not available, therefore we cannot employ a formal test of a scenario against the observed record.^5^ Future development of scenarios could apply the methods proposed by Müller and Watson [16] to create approximate probabilistic projections of socio-economic paths. Müller and Watson introduce an approach to construct prediction sets for long-horizon forecasts of scalar variables, and in their application they consider projections of US economic time series over horizons ranging from 10 to 75 years.

The adequacy of IPCC projections has been discussed previously (see Refs. [7], [26], [22]); however, the focus has remained predominately on levels rather than growth rates. This is less meaningful given the aforementioned variation in initial values across scenarios. Additionally, the focus remained primarily on CO_2_ emissions and not the wider socio-economic variables or crucially, emission intensity.^6^ Due to a lack of data, few studies investigated the performance of scenarios inclusive of the year 2010. Indeed, earlier studies, such as Raupach et al. [21] find fossil-fuel CO_2_ emissions to be accelerating over the 1990s and early 2000s. Nevertheless, with IPCC projections only available at a decadal interval, the growth over the 2000s could not be assessed. Additional earlier studies using data up to 2009 find the level of CO_2_ emissions to fall within the SRES range [26] and using within model group averages, to lie on the upper end of the 2000 scenarios [22]. However, wider socio-economic variables, emission intensity, or the longer projections (published in 1992) are not considered. On the physical-science side, a first assessment of an early probabilistic temperature forecast conditioned on a single scenario finds that observed global mean temperatures fall within the predicted interval [2]. We provide a similar analysis by assessing the accuracy of the underlying socio-economic scenarios. This equally permits an assessment of whether the single scenario chosen in the temperature forecast analysis by Allen et al. [2] not only matches observed temperatures but also the socio-economic evolution which determines the anthropogenic component in changes in climate.

## 2 Methods

Due to variations in initial values of the socio-economic scenarios we calculate growth rates and compare these to the observed growth rates of population, fossil-fuel CO_2_ emissions, real GDP (based on market exchange rates to be consistent with the units used in the scenarios), and emission intensity. We compute the annual average growth over decades (referred to as decadal growth rate throughout) for both global observed and projected values of the socio-economic series.

A method is needed to disaggregate global differences in growth rates to country-by-country contributions to assess what drives any observed global discrepancy: we downscale scenario values (relying on two different downscaling approaches) to individual countries and decompose the aggregate (global) difference between observed and projected growth rates into country contributions. The proposed decomposition can be applied to any downscaling method chosen. It is worth highlighting that the downscaling is not used to assess the accuracy of the scenarios but rather to explain the observed global differences.

### 2.1 Downscaling the SRES projections

To disaggregate the global difference in projected and observed growth rates, we require country-by-country contributions to this global difference. We use two downscaling approaches. First we apply a simple linear downscaling procedure, and second, we use the downscaled values provided by van Vuuren et al. [27].

We emphasize that this is done to disaggregate the difference between global projected and observed growth rates and not to assess the accuracy of the scenarios by comparing them to country-observed values. This allows us to determine if the aggregate difference is driven by a subset of countries in particular, or if the contribution to the global error is similar across countries.

In linear downscaling, individual countries are assigned their corresponding regional growth rates based on the four regions defined in the IPCC SRES projections. These regions are: REF – countries undergoing economic reform, the OECD90 region, ASIA, and ALM – Africa and Latin America.^7^ Each region in the SRES projections has a common growth rate across all countries. For level reconstructions the projected growth rates are applied to the individual country's^8^ observed values in 2000 ([3], [25]; [28]). Linear downscaling is chosen here as the first method for transparency as it does not require additional assumptions on regional convergence and preserves the differences in initial values. While linear downscaling does not account for heterogeneity of growth rates within regions, the difference in observed initial values implies there will be no convergence in the level of the variables within the regions.

To assess the robustness of our results to the downscaling method, we also use the van Vuuren et al. [27] downscaled SRES data set^9^ based on assumptions of gradual regional convergence as an alternative to linear downscaling. Over long time scales (e.g. the climate projections up to 2100) we expect the values of different downscaling methods to diverge due to compounding. However, over the drastically shorter time period considered here (2000–2010 for the SRES scenarios), the type of downscaling approach has little effect on the final values (see Section 4).

### 2.2 Decomposition of aggregate growth rates

We are interested in explaining the difference between observed global ($G_{t})$ and projected global growth rates (${\hat{G}}_{t}$). For this we investigate which countries are most important in explaining the aggregate difference. We use individual country level data from 2000 to 2010 to calculate observed country-level growth rates. For each scenario we use the projected and subsequently downscaled country-level growth rates. To attribute the discrepancies in global growth rates to individual contributions to those at country-level, we propose the following approach to decompose aggregate growth rates.^10^

#### 2.2.1 Decomposing aggregate growth rates to country-by-country contributions

Let $Y_{t}=\sum_{j}Y_{t}^{j}$ denote aggregate GDP over countries *j*, and let$\;Z_{t}=\frac{C_{t}}{Y_{t}}$ denote aggregate fossil fuel CO_2_ emissions per GDP, where aggregate fossil fuel emissions $C_{t}=\sum_{j}C_{t}^{j}$ are summed over countries *j*. Each country *j's* fossil fuel CO_2_ emissions per GDP is defined as $Z_{t}^{j}=\frac{C_{t}^{j}}{Y_{t}^{j}}$. The corresponding aggregate ($G_{t})$ and individual ($G_{t}^{j}$) growth rates are given by $G_{t}=\frac{Z_{t}-Z_{t-1}}{Z_{t-1}}$ and $G_{t}^{j}=\frac{Z_{t}^{j}-Z_{t-1}^{j}}{Z_{t-1}^{j}}$ respectively.

The aggregate growth rate can then be re-expressed as:

(1) $$G_{t}=\frac{Z_{t}-Z_{t-1}}{Z_{t-1}}=\frac{1}{Z_{t-1}}\left(\frac{\sum_{j}C_{t}^{j}}{Y_{t}}-\frac{\sum_{j}C_{t-1}^{j}}{Y_{t-1}}\right)$$

As shown in the supplementary material this can be simplified to:

(2) $$G_{t}=\left(\sum_{j}\frac{C_{t-1}^{j}}{Y_{t-1}^{j}}\left[\frac{Y_{t}^{j}}{Y_{t}}\left(1+G_{t}^{j}\right)-\frac{Y_{t-1}^{j}}{Y_{t-1}}\right]\right)\frac{1}{Z_{t-1}}=\sum_{j}\frac{Z_{t-1}^{j}}{Z_{t-1}}\left[\frac{Y_{t}^{j}}{Y_{t}}-\frac{Y_{t-1}^{j}}{Y_{t-1}}+G_{t}^{j}\frac{Y_{t}^{j}}{Y_{t}}\right]$$

(3) $$G_{t}=\sum_{j}\frac{Z_{t-1}^{j}}{Z_{t-1}}\left[\Delta \frac{Y_{t}^{j}}{Y_{t}}+G_{t}^{j}\frac{Y_{t}^{j}}{Y_{t}}\right]=\sum_{j}d_{j}$$

where we define the individual country contribution as $d_{j}=$ $\frac{Z_{t-1}^{j}}{Z_{t-1}}\left[\Delta \frac{Y_{t}^{j}}{Y_{t}}+G_{t}^{j}\frac{Y_{t}^{j}}{Y_{t}}\right]$, where the term $\Delta \frac{Y_{t}^{j}}{Y_{t}}=\frac{Y_{t}^{j}}{Y_{t}}-\frac{Y_{t-1}^{j}}{Y_{t-1}}$ captures the change in the proportion of country *j*'s GDP relative to total GDP. The individual contributions $d_{j}$ can be decomposed into an emission intensity “growth rate effect” and a “GDP effect”, which is the change in country *j's* GDP relative to total GDP:

(4) $$d_{j}=\frac{Z_{t-1}^{j}}{Z_{t-1}}\left[\Delta \underset{GDP\;Effect}{\underset{︸}{\frac{Y_{t}^{j}}{Y_{t}}}}+\underset{Growth\;Rate\;Effect}{\underset{︸}{G_{t}^{j}\frac{Y_{t}^{j}}{Y_{t}}}}\right]$$

If the ratio of the particular country's GDP to global GDP is unchanged $\left(\Delta \frac{Y_{t}^{j}}{Y_{t}}=0\right)$, then the only contribution to the overall growth rate is derived from the growth rate effect: $\frac{Z_{t-1}^{j}}{Z_{t-1}}\;G_{t}^{j}\frac{Y_{t}^{j}}{Y_{t}}$. Whether the contribution to the overall growth rate is positive or negative depends on the change in the ratio of a country's GDP relative to the global GDP, and a country's growth rate in emissions per GDP, $G_{t}^{j}$ scaled by the share of the country's GDP relative to the global GDP.

#### 2.2.2 Decomposing the difference between observed and projected aggregate growth rates

We compare the observed growth rate ($G_{t})$ against a scenario predicted one (${\hat{G}}_{t}$) using the same decomposition procedure as in Section 2.2.1. The difference between observed and predicted growth rates can be attributed to disaggregated country contributions and further into relative GDP change and emission intensity growth rate effects:

(5) $$G_{t}-{\hat{G}}_{t}=\;\sum_{j}d_{j}-\;\sum_{j}{\hat{d}}_{j}\;$$

(6) $$G_{t}-{\hat{G}}_{t}=\sum_{j}\underset{GDP\;Effect}{\underset{︸}{\left(\frac{Z_{t-1}^{j}}{Z_{t-1}}\left[\Delta \frac{Y_{t}^{j}}{Y_{t}}\right]-\frac{{\hat{Z}}_{t-1}^{j}}{{\hat{Z}}_{t-1}}\left[\Delta \frac{{\hat{Y}}_{t}^{j}}{{\hat{Y}}_{t}}\right]\right)}}+\underset{Growth\;Rate\;Effect}{\underset{︸}{\left(\frac{Z_{t-1}^{j}}{Z_{t-1}}G_{t}^{j}\left[\frac{Y_{t}^{j}}{Y_{t}}\right]-\frac{{\hat{Z}}_{t-1}^{j}}{{\hat{Z}}_{t-1}}{\hat{G}}_{t}^{j}\left[\frac{{\hat{Y}}_{t}^{j}}{{\hat{Y}}_{t}}\right]\right)}}$$

The decomposition proposed here is independent of the downscaling method applied, and we present the results of applying this decomposition to both linearly downscaled, as well as the van Vuuren et al. downscaled data in Section 4.

## 3 Data

IPCC scenario data are obtained from the IPCC Data Distribution Center [9]. Observed fossil-fuel emissions^11^ are available at global and national level [3]. Global and national population data are obtained from the UN Population Division [25]. GDP on global and national scale is measured in 1990 market exchange rate converted USD [28] to be consistent with SRES measures.

## 4 Results

### 4.1 Comparing global emission intensity growth to global scenario projections

The world has seen growth in CO_2_ emission intensity over the 2000s not envisaged by any of the main scenarios (shown in Fig. 1 and Table 1 as percentage change in tonnes of carbon per $1,000, panel b). While all main socio-economic scenarios projected declining emission intensity, the observational record shows that emission intensity was in fact rising. The average decadal growth rate in emission intensity over the 2000s of 0.37% per year considerably exceeded even the closest marker scenario growth rate (A1), which projected a decline of −0.1%. The remaining main SRES and IS92 scenarios project declines ranging from −0.3% to −1.75%. The plot of levels (rather than growth rates) of emissions intensity hides this discrepancy between scenarios and observations (see Fig. 1, panel a). In fact, the level projections only appear to match the observations closely due to the mismatch in starting values. These results are consistent for fossil-fuel CO_2_ emissions per GDP per capita – observed growth rates in per capita terms exceed all projected IS92 and SRES marker scenarios (see Supplementary Fig. S9). Following a consistent decline in emission intensity over the 1990s, the world did not see a continued decline over the 2000s. However, observations from the 2010s provide evidence that there may have been a recent turn-around (see Ref. [10]).

**Table 1:** Scenario-Projected and Observed Growth in Global Emission Intensity (Fossil-Fuel CO_2_ emissions per GDP). Values correspond to annually-averaged decadal growth rates, also shown in Fig. 1, Fig. 2.

		1990–2000	2000–2010
SRES	A1 AIM		−0.10%
	A2 ASF		−0.31%
	B1 IMAGE		−1.17%
	B2 MESSAGE		−1.51%
	A1F1		−0.97%
	A1T		−1.20%
IS 92	IS 92A	−1.17%	−1.17%
	IS 92B	−1.43%	−1.37%
	IS 92C	−1.48%	−1.42%
	IS 92D	−1.78%	−1.75%
	IS 92E	−1.17%	−1.28%
	IS 92F	−0.89%	−1.00%
Observed		−1.66%	0.37%

**Fig. 2:**
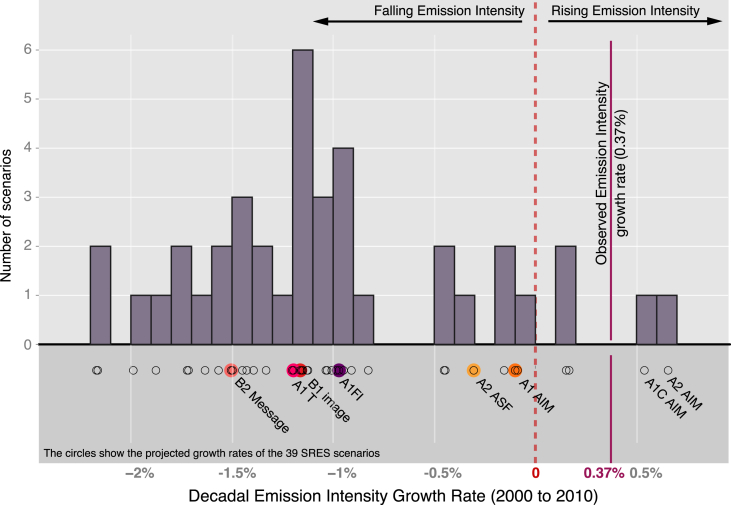
Histogram of projected global emission intensity decadal growth rates over the 2000s for all SRES scenarios and observed global decadal growth rate (solid purple). The 6 main SRES scenarios are shown in colour, circles show projected growth rates across all 39 SRES scenarios. Observed positive decadal growth rates exceed all main scenario projections over the 2000s, which envisaged a decline in emission intensity rather than an increase. (For interpretation of the references to colour in this figure legend, the reader is referred to the web version of this article.)

Fig. 2 expands the set of scenarios considered beyond the main scenarios by plotting the 2000s growth rates across the full set of SRES scenarios created.^12^ Consistent with the results relative to the main scenarios, observed growth rates exceed projected growth rates. Only 2 out of the 39 scenarios are higher than observed emission intensity growth for the time period considered, and only 4 out of 39 projections suggest an increase of emission intensity (positive growth rate) rather than a decline.

### 4.2 Decomposing global differences to country-by-country contributions

**Fig. 3:**
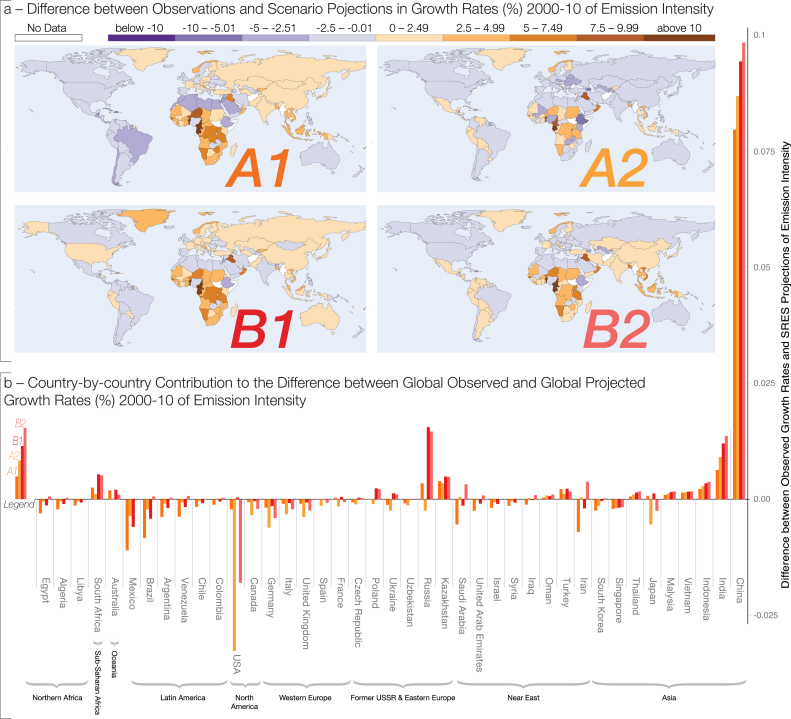
Decomposition of the global difference between global observed and projected emission intensity using country-by-country downscaled difference between observed and projected decadal growth rates in emission intensity for SRES marker projections over 2000–2010. The four world maps in panel a show the difference between observed and projected growth rates in fossil-fuel CO_2_ emissions per GDP (underestimated growth rates in brown tones, overestimated in purple). Panel b graphs the country-by-country contribution to the difference between global observed and global projected growth rates in emission intensity. The primary contribution to the differences in global growth rates relative to SRES projections stems from unanticipated changes in Asia. (For interpretation of the references to colour in this figure legend, the reader is referred to the web version of this article.)

To investigate the systematic discrepancy in global emission intensity growth rates between observations and scenarios, we decompose the aggregate difference using methods in Section 2 to a country-level for four of the SRES projections (Fig. 3, panel *a*). These scenarios were chosen as they are the core markers, with A1 and B1 indicating the scenarios with the smallest and highest global deviation from observations respectively. Results using the van Vuuren et al. [27] downscaled data are reported in the supplementary material S6. The apparent regional differences are consistent across all four marker scenarios. The observed growth rates of fossil-fuel CO_2_ emission intensity in Sub-Saharan African and South Asian countries exceed those of the downscaled scenario growth rates as shown in the map in Fig. 3. Across all scenarios, growth rates observed in Latin America are predominantly below scenario projections.

To explain what drives the global difference between observed and projected growth rates, we quantify the country-by-country contribution to this global difference. The importance of the country in terms of the level of GDP and emissions intensity can be obtained through their relative weighting using the decomposition in Section 2.2. While Sub-Saharan African countries exhibit the highest deviations from regional-scenario projections (Fig. 2, panel a), their overall contribution to the global difference in growth rates is small (Fig. 2, panel *b*). When weighted according to their contribution to global emission intensity, it becomes apparent that unforeseen changes in China, Russia, and wider Asia account for the dominant share of the difference between global observed and SRES projected growth rates (Fig. 2, panel *b* using linear downscaling, and Fig. S10 using downscaled data from Ref. [27]). It is important to emphasize that this is *relative to the projections* and that the emission intensity in China and Russia actually declined over the 2000s (see Supplementary Fig. S8). The discrepancy is primarily driven by unanticipated growth in GDP (the ‘GDP effect’ in Equation (6)) in Asia.^13^ Our study over this longer time-span provides evidence for the continuation of the trends found by Raupach et al. [21]. The results are not driven by the choice of the downscaling method, repeating the analysis with the van Vuuren et al. [27] downscaled SRES results which assume regional convergence confirms the main results.^14^ Rapid growth in Asia was not anticipated in most projections, including the World-Energy Outlook [24], the Federal Reserve, or the Consensus Economics forecasts: even over much shorter horizons of one quarter to one-and a half years, GDP forecasts produced by the Federal Reserve exhibit the highest forecast errors for China over the 2000s [5]. On a decadal scale, observed Chinese GDP growth exceeds the Consensus Economics [4] forecasts by 7%, a similar magnitude as SRES projections, which are exceeded by 2–11%. IPCC projections – despite not being forecasts per-se and while considering much longer time horizons – do not appear systematically worse than alternative projections for the time period.

### 4.3 Scenario-tracking across additional socio-economic variables

More broadly, we assess if there is a single scenario that observations are tracking more closely than others. This is to evaluate if one of the scenario groups (ranging from fossil-fuel intense convergent growth, to ecologically-friendly divergence) provides a comprehensive explanation for socio-economic developments over the 1990s and 2000s. We assess which scenario is closest to the observed record as measured by the smallest proportional deviation from observed levels, and lowest absolute difference to growth rates. No single scenario uniquely dominates other scenarios when assessed against the observed variables over both time intervals (see Fig. 1, Fig. 3, here, and Supplementary Figs. S1–S6, and Table S1). Notably, earlier IS92 scenarios are not systematically further away from observations than later SRES projections. Given the inertia of population dynamics, it is not surprising that population projections exhibit the lowest deviations from observations (Supplementary Fig. S2) (see also [15]). Overall, no single ‘story-line’ of the scenarios captures the observed socio-economic development more closely than others during the 1990s and 2000s.^15^

**Fig. 4:**
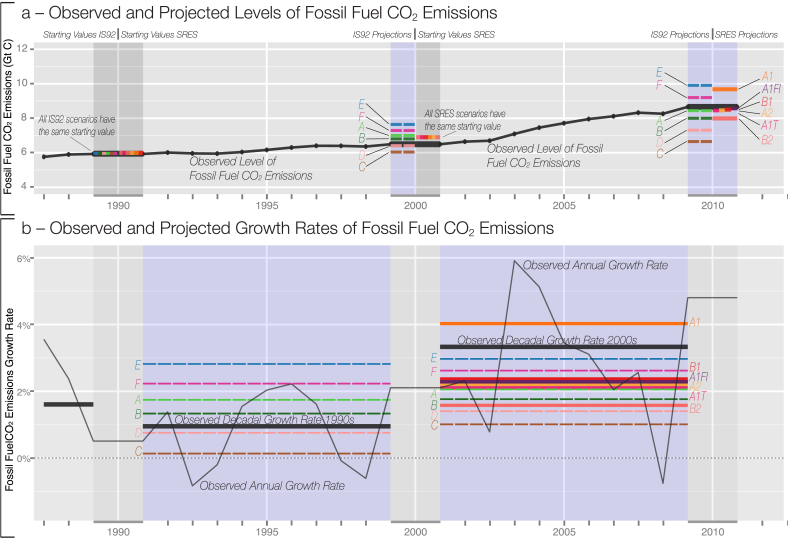
Observed (black) and projected (colour) global fossil-fuel CO2 emissions in levels (a) and growth rates (b). Panel a graphs global observed emissions together with decadal IS92 and main SRES marker projections. Panel b shows observed annual growth rates together with observed decadal growth rates over both decades. Observed decadal growth rates exceed nine out of ten scenario projections over the 2000s. (For interpretation of the references to colour in this figure legend, the reader is referred to the web version of this article.)

The 1992 A scenario is the closest IS92 projection in levels of CO_2_ emissions, thus supporting the choice of this scenario for the global mean temperature forecast in Allen et al. [2]; as the level of CO_2_ emissions is most relevant for a global mean temperature forecast. There is no forecast failure of global mean temperature in Allen et al., as the levels of observed CO_2_ emissions are closely matched by the chosen scenario. However, over the 2000s, nine of the ten projections underestimated decadal growth in fossil-fuel CO_2_ emissions (Fig. 4, in GtC, panel b) similarly to emission intensity. While temperature forecasts are not rejected in the short-term, we may see a long-term increase in divergence of temperatures from early scenario values, based on the under-projection of growth rates in both CO_2_ emission and emission intensity. Evidence for this can already be seen through the accelerating accumulation of concentrations of CO_2_ in the atmosphere. The growth between 2012 and 2013 was the highest observed since 1984 [29], and is also supported through the future outlook on CO_2_ emissions by Friedlingstein et al. [6].

## 5 Conclusion

An assessment of socio-economic projections can potentially reduce the uncertainty about future climate change by highlighting systematic discrepancies and biases in existing scenarios. Evaluation of the IPCC projections against the observed record over two decades requires careful analysis of the growth rates due to substantial differences in initial values in levels across the different projections. For future development it is important that scenarios are designed with consistent starting values (despite intrinsic uncertainties about observations) to enable comparisons in levels as well as growth rates, as the RCPs now do.

Our analysis of growth rates revealed that global emissions intensity growth exceeded 37 of all 39 SRES scenarios – including all 6 main scenarios – over the decade 2000–2010. To assess the role of individual countries, we show how aggregate differences in growth rates can be decomposed into individual contributions when down-scaling is necessary due to a coarser resolution of the projected values relative to observational data. Applying the proposed decomposition, we find the global difference in emission intensity growth rates to be driven by unanticipated GDP growth in Asia and Eastern Europe. Our scenario evaluation shows the impact of local unforeseen shifts in growth, and demonstrates that observations over the 1990s and 2000s do not track any single scenario family more closely than others.

Overall, the apparent under-projection of emission intensity raises concerns about achieving sustainable energy production. There appears to be little evidence of a de-coupling of growth from emissions during the 2000s, though the outlook appears a little brighter as data for 2014 and 2015 show an ‘unexpected’ recent de-coupling of growth from emissions [10].

More generally, our results raise the question of how scenarios and forecasts should be used to guide policy in the presence of shifts and instabilities. Given the impact of shifts in single countries (as identified using the growth decomposition) additional focus could be shifted onto creating projections on a more local rather than global scale. On the climate policy side, Otto et al. [18] and Millar et al. [14] make a convincing argument for linking climate mitigation goals explicitly to climate responses to be robust to even the worst-case scenarios.

## Footnotes

2 The RCPs are scenarios of concentrations, corresponding socio-economic scenarios are available as the shared socio-economic pathways (SSPs).

3 We investigate population projections and fossil-fuel CO_2_ emissions per GDP per capita which combines all three socio-economic scenarios in supplementary material 6.5.

4 The six 'illustrative scenarios' [13] consist of the four core marker scenarios (A1 AIM, A2 ASF, B1 IMAGE, B2 MESSAGE) and the additional scenarios of A1FI (A1G MiniCAM) and A1T MESSAGE. In total there are 40 scenarios in the SRES, out of the 40, only two A2 AIM (0.65), A1 AIM (0.54) show higher than observed growth rates, and these two are not the ‘illustrative’ scenarios or marker scenarios. The next highest 0.16, then 0.14, all others are negative.

5 Equally a test of the average annual growth rate against scenario growth rates provides little insight since the assumption that the observed growth rate in a particular year is equal to the scenario growth rate is not made in the scenarios.

6 Some studies focus on a subset of projected series, for example [19] assess the IPCC coal-production projections.

7 While the IPCC regional list does not include the United Kingdom, we include it within the OECD90 region.

8 Starting values in 2000 are chosen to be able to include a wide range of Eastern European countries for which no observations in 1990 are available.

9 [27] provide the downscaled values for the IMAGE model groups, of which B1 is part of the set of 'illustrative marker scenarios' recommended by Ref. [13].

10 The derivations are shown in detail in the supplementary material.

11 The measure of CO_2_ accounts for fossil-fuel emissions to match the measure used in the IPCC projections.

12 There is a total of 41 SRES scenarios, GDP projections are available for 39 of these.

13 It is not primarily changes in CO_2_ emissions in China but rather the increase in Chinese GDP relative to world total GDP that account for China's contribution to the difference in aggregate growth rates. As we show in Supplementary Section 6.2, real GDP in China relative to aggregate global GDP increased more than anticipated in the scenarios, and drives the aggregate discrepancy in emission intensity growth rates. See also Supplementary Section S3.3 for a decomposition of the difference into the GDP and growth-rate effects.

14 Fig. S10 shows that the main difference is driven by unanticipated high growth in Asia, a pattern consistent with Fig. 3 which uses linear downscaling.

15 The details of the best fitting scenarios of each main socio-economic projection are listed in the supplementary material for both levels and growth rates.
