# Supplementary material for: Carbon dioxide emission-intensity in climate projections: Comparing the observational record to socio-economic scenarios
Source: Energy (Oxf). 2017 Sep 15;135:718–25. doi: 10.1016/j.energy.2017.06.119 (PMC5625523; doi:10.1016/j.energy.2017.06.119)

Supplementary Material

Carbon Dioxide Emission-Intensity in Climate Projections: Comparing the Observational Record to Socio-Economic Scenarios

S1 Observed Socio-Economic Indicators and Scenario Projections

Figure S1: Global real GDP in levels in 1990 USD (top) and growth rates (bottom). Observed are shown in black, projections in colour.

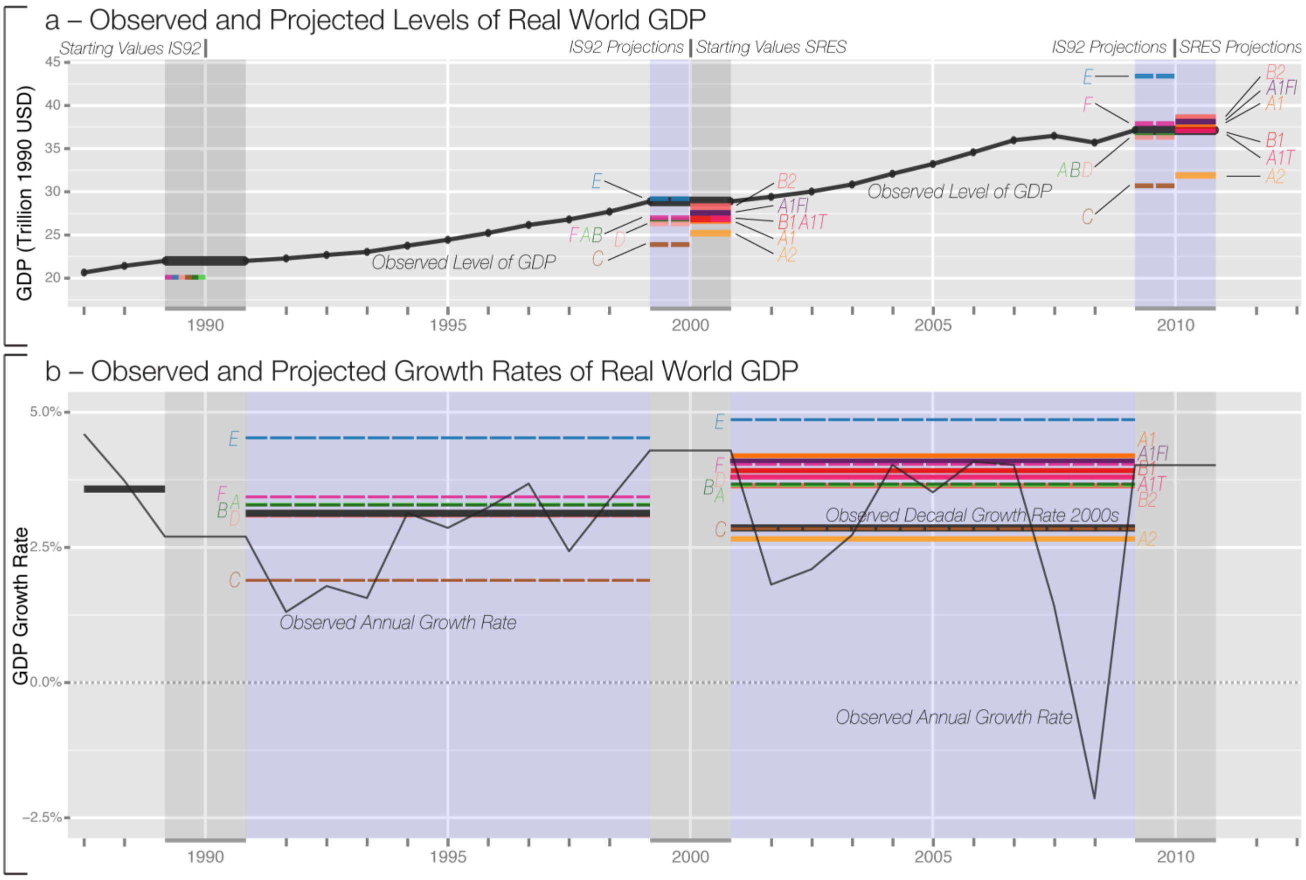

Figure S2: Global population in levels (top) and growth rates (bottom). Observed are shown in black, projections in colour.

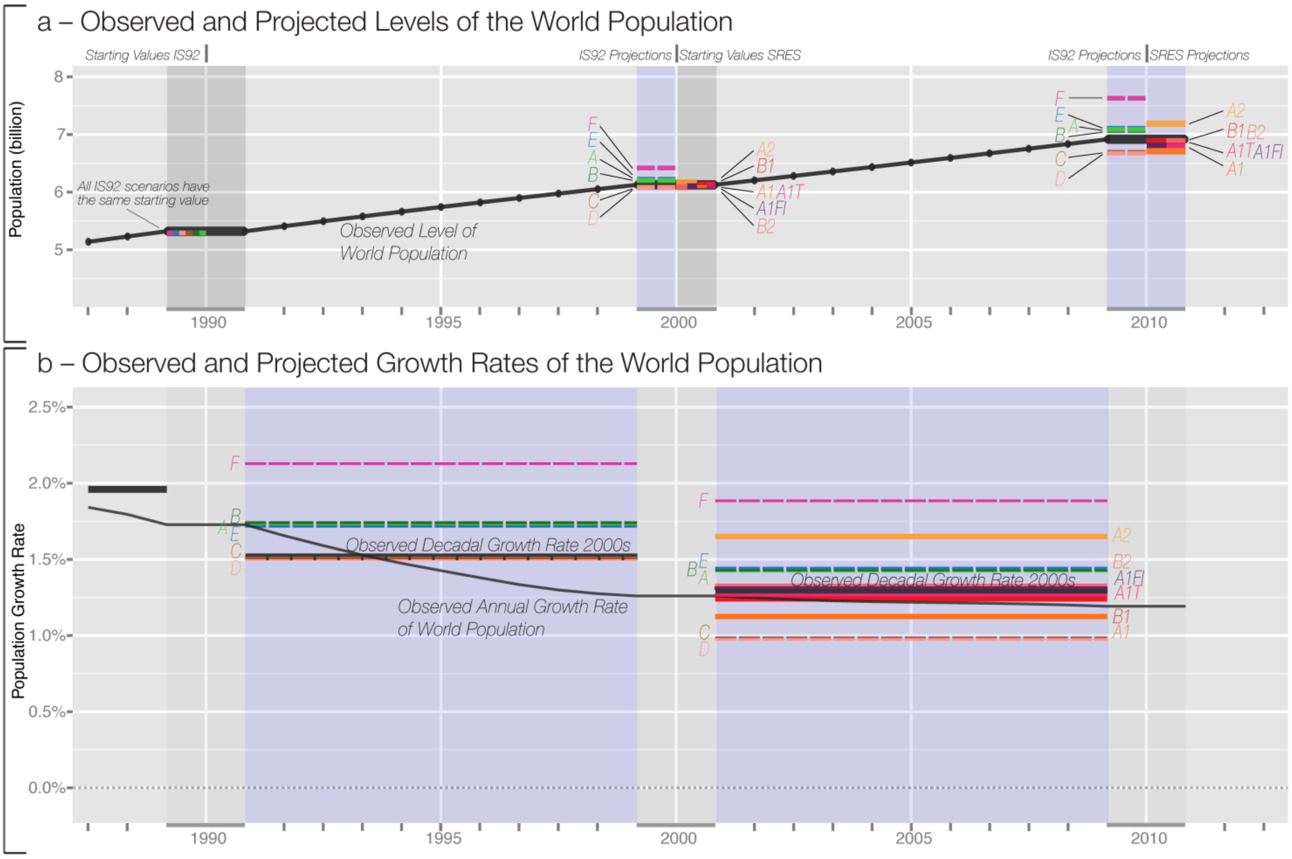

S2 Relative Scenario Performances

Table S1 lists the most accurate scenario based both on levels and growth rates for both 1990s and 2000s. Accuracy here is measured by the smallest absolute percentage deviation in levels, and the smallest absolute deviation in growth rates.

Table S1: Closest SRES (red) and IS92 (blue) scenarios for 1990-2000 and 2000-2010 based on proportional deviation from observed levels and absolute difference from observed growth rates. Deviations are shown in parentheses.

	**GDP**		**Population**		**Fossil Fuel CO_2**		**CO_2 Intensity**	
Time	Level	Growth	Level	Growth	Level	Growth	Level	Growth
1990-2000	**E (0.011)**	**D (-0.045)**	**C/D (0.013)**	**C/D (0.206)**	**D (-0.012)**	**D (-0.196)**	**D (0.084)**	**D (-0.122)**
2000-2010	**B (-0.017)**<br>**B1 (0.004)**	**C (-0.015)**<br>**A2 (-0.201)**	**A/B/E (0.025)**<br>**B1 (-0.003)**	**A/B/E (0.137)**<br>**A1FI (-0.016)**	**A1FI (-0.001)**<br>**B1 (-0.018)**	**E (-0.361)**<br>**A1 (0.697)**	**E (-0.008)**<br>**B1 (-0.023)**	**F (-1.375)**<br>**A1 (-0.474)**

Figure S3: CO_2 Intensity Differences to Observed Levels (top) and Growth Rates (bottom) over 1990-2000 (left) and 2000-2010 (right)

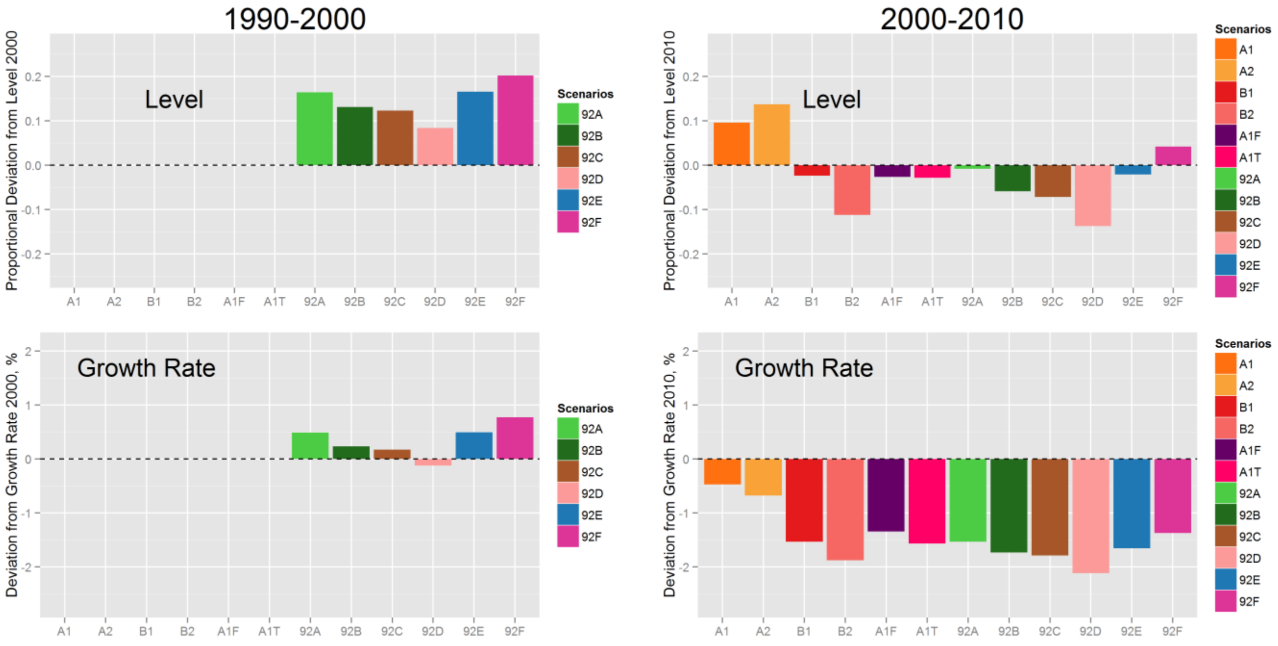

Figure S4: GDP Differences to Observed Levels (top) and Growth Rates (bottom) over 1990-2000 (left) and 2000-2010 (right)

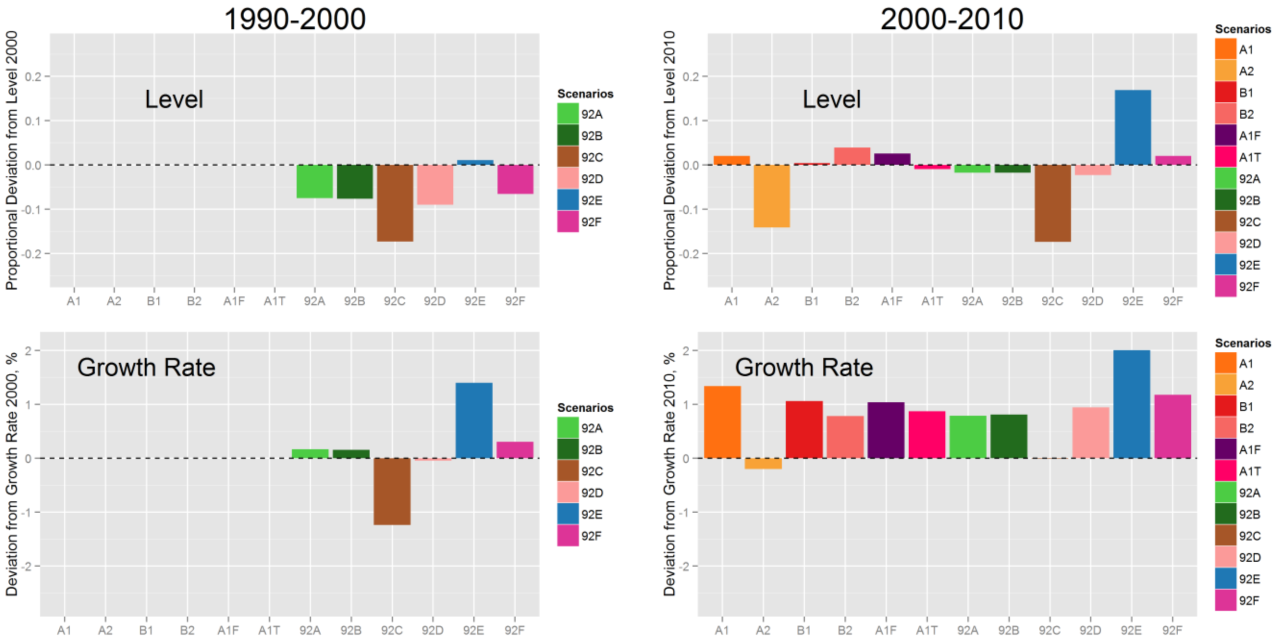

Figure S5: Population Differences to Observed Levels (top) and Growth Rates (bottom) over 1990-2000 (left) and 2000-2010 (right)

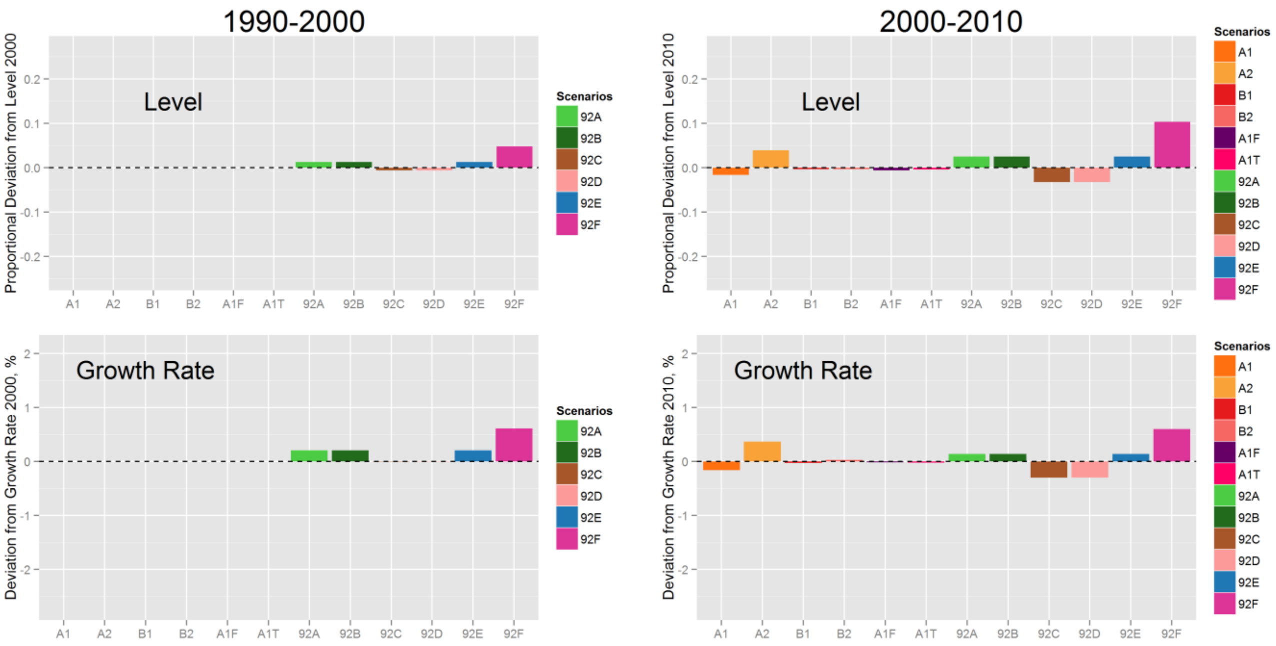

Figure S6: Fossil Fuel CO_2 Differences to Observed Levels (top) and Growth Rates (bottom) over 1990-2000 (left) and 2000-2010 (right

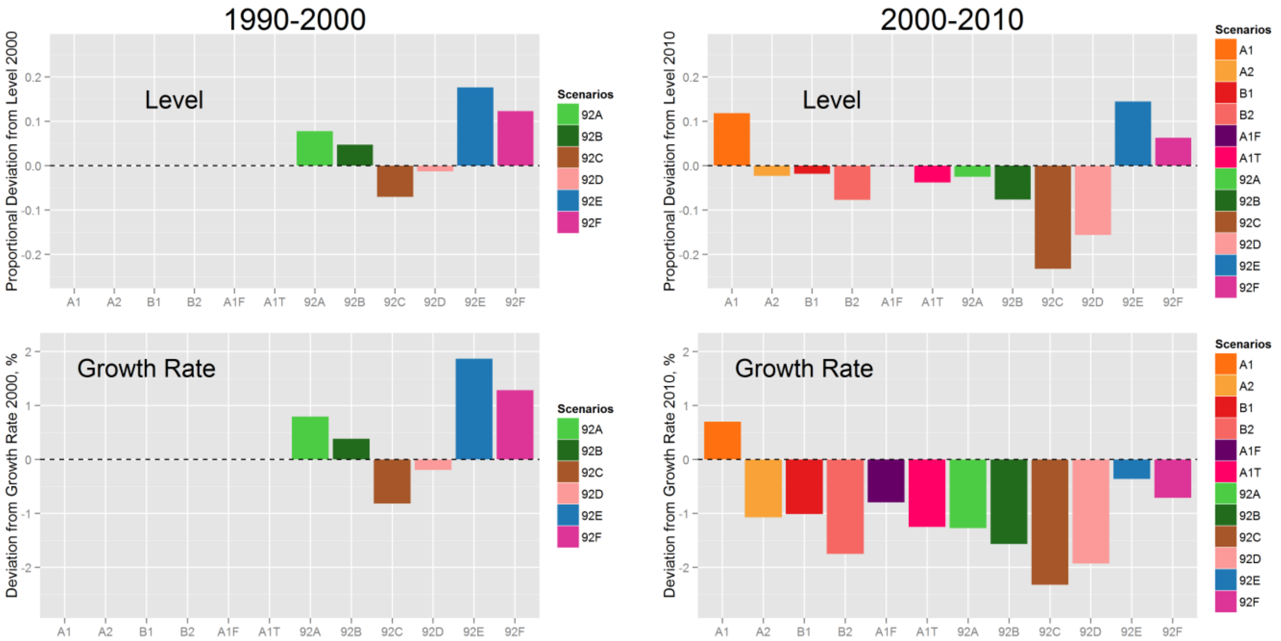

S3: Details of the Growth Decomposition

S3.1 Decomposing Aggregate Growth Rates

Let $Y_t = \sum_j Y_t^j$ denote aggregate GDP over countries *j*, and let $Z_t = \frac{C_t}{Y_t}$ denote aggregate fossil fuel CO_2 emissions per GDP, where aggregate fossil fuel emissions $C_t = \sum_j C_t^j$ are summed over countries *j*. Each country *j's* fossil fuel CO_2 emissions per GDP is defined as $Z_t^j = \frac{C_t^j}{Y_t^j}$. The corresponding aggregate (G_t) and individual (G_t^j) growth rates are given by $G_t = \frac{Z_t - Z_{t-1}}{Z_{t-1}}$ and $G_t^j = \frac{Z_t^j - Z_{t-1}^j}{Z_{t-1}^j}$ respectively.

The aggregate growth rate can then be re-expressed as:

$$G_t = \frac{Z_t - Z_{t-1}}{Z_{t-1}} = \frac{1}{Z_{t-1}}\left(\frac{\sum_j C_t^j}{Y_t} - \frac{\sum_j C_{t-1}^j}{Y_{t-1}}\right) \tag{1}$$

From above we use that the equation for the individual growth rate can be re-arranged to yield:

$$G_t^j \frac{C_{t-1}^j}{Y_{t-1}^j} = \frac{C_t^j}{Y_t^j} - \frac{C_{t-1}^j}{Y_{t-1}^j} \tag{2}$$

This provides an expression for country *j*'s fossil fuel emissions C_t^j:

$$C_t^j = (1 + G_t^j) C_{t-1}^j \frac{Y_t^j}{Y_{t-1}^j} \tag{3}$$

Therefore we can write the aggregate growth rate as a function of disaggregate growth rates by substituting for C_t^j in:

$$G_t = \left(\sum_j \frac{1}{Y_t} C_t^j - \frac{1}{Y_{t-1}} C_{t-1}^j\right) \frac{1}{Z_{t-1}} \tag{4}$$

This can be simplified to:

$$G_t = \left(\sum_j \frac{C_{t-1}^j}{Y_{t-1}^j}\left[\frac{Y_t^j}{Y_t}(1 + G_t^j) - \frac{Y_{t-1}^j}{Y_{t-1}}\right]\right)\frac{1}{Z_{t-1}} = \left(\sum_j \frac{Z_{t-1}^j}{Z_{t-1}}\left[\frac{Y_t^j}{Y_t} - \frac{Y_{t-1}^j}{Y_{t-1}} + G_t^j \frac{Y_t^j}{Y_t}\right]\right) \tag{5}$$

$$G_t = \left(\sum_j \frac{Z_{t-1}^j}{Z_{t-1}}\left[\Delta \frac{Y_t^j}{Y_t} + G_t^j \frac{Y_t^j}{Y_t}\right]\right) = \left(\sum_j d_j\right) \tag{6}$$

Where $d_j = \frac{Z_{t-1}^j}{Z_{t-1}}\left[\Delta \frac{Y_t^j}{Y_t} + G_t^j \frac{Y_t^j}{Y_t}\right]$ and the term $\Delta \frac{Y_t^j}{Y_t} = \frac{Y_t^j}{Y_t} - \frac{Y_{t-1}^j}{Y_{t-1}}$ captures the change in the proportion of country *j*'s GDP relative to total GDP. This yields the aggregate growth rates as the sum of the individual components *j* and allows for a decomposition of the aggregate growth rates into individual contributions d_j.

The individual contributions can be decomposed further into an "emission intensity growth rate effect" and a "relative change in GDP effect":

$$d_j = \frac{Z_{t-1}^j}{Z_{t-1}} \left[\underbrace{\Delta \frac{Y_t^j}{Y_t}}_{GDP\ Effect} + \underbrace{G_t^j \frac{Y_t^j}{Y_t}}_{Growth\ Rate\ Effect} \right] \tag{7}$$

If the ratio of the particular country's GDP to global GDP is unchanged ($\Delta \frac{Y_t^j}{Y_t} = 0$), then the only contribution to the overall growth rate is derived from the growth rate effect: $\frac{Z_{t-1}^j}{Z_{t-1}} G_t^j \frac{Y_t^j}{Y_t}$. Whether the contribution to the overall growth rate is positive or negative therefore depends on the change in the ratio of a country's GDP relative to the global GDP, and a country's growth rate in emissions per GDP, G_t^j scaled by the weight of the country's GDP relative to the global GDP.

S3.2 Decomposing the Difference between Observed and Projected Aggregate Growth Rates

When comparing an observed growth rate (G_t) against a scenario predicted growth rate ($\hat{G}_t$), using the same decomposition procedure as above, the difference between observed and predicted growth rates can be attributed to disaggregated country contributions, and further into relative GDP change and emission intensity growth rate effects. Given the summability, simple regional aggregates can be considered as well as country-level disaggregation. These are plotted in Figure 2 (panel *b*) for individual contributions to the overall difference in growth rates exceeding 0.1%.

$$G_t - \hat{G}_t = \sum_j d_j - \sum_j \hat{d}_j \tag{8}$$

$$G_t - \hat{G}_t = \sum_j \frac{Z_{t-1}^j}{Z_{t-1}} \left[\Delta \frac{Y_t^j}{Y_t} + G_t^j \frac{Y_t^j}{Y_t} \right] - \sum_j \frac{\hat{Z}_{t-1}^j}{\hat{Z}_{t-1}} \left[\Delta \frac{\hat{Y}_t^j}{\hat{Y}_t} + \hat{G}_t^j \frac{\hat{Y}_t^j}{\hat{Y}_t} \right] \tag{9}$$

$$G_t - \hat{G}_t = \sum_j \underbrace{\left(\frac{Z_{t-1}^j}{Z_{t-1}} \left[\Delta \frac{Y_t^j}{Y_t} \right] - \frac{\hat{Z}_{t-1}^j}{\hat{Z}_{t-1}} \left[\Delta \frac{\hat{Y}_t^j}{\hat{Y}_t} \right] \right)}_{GDP\ Effect} + \underbrace{\left(\frac{Z_{t-1}^j}{Z_{t-1}} G_t^j \left[\frac{Y_t^j}{Y_t} \right] - \frac{\hat{Z}_{t-1}^j}{\hat{Z}_{t-1}} \hat{G}_t^j \left[\frac{\hat{Y}_t^j}{\hat{Y}_t} \right] \right)}_{Growth\ Rate\ Effect} \tag{10}$$

S3.3 Total Growth Rate Decomposition into GDP and pure Emission Intensity Growth Rate Effect

Country-by-country contributions to the difference in observed world growth rates and projected growth rates can be further decomposed into a GDP effect and a growth rate effect using the results from section 5.2:

$$G_t - \hat{G}_t = \sum_j \underbrace{\left(\frac{Z_{t-1}^j}{Z_{t-1}} \left[\Delta \frac{Y_t^j}{Y_t} \right] - \frac{\hat{Z}_{t-1}^j}{\hat{Z}_{t-1}} \left[\Delta \frac{\hat{Y}_t^j}{\hat{Y}_t} \right] \right)}_{GDP\ Effect} + \underbrace{\left(\frac{Z_{t-1}^j}{Z_{t-1}} G_t^j \left[\frac{Y_t^j}{Y_t} \right] - \frac{\hat{Z}_{t-1}^j}{\hat{Z}_{t-1}} \hat{G}_t^j \left[\frac{\hat{Y}_t^j}{\hat{Y}_t} \right] \right)}_{Growth\ Rate\ Effect}$$

Figure S7 graphs the difference in growth rate decomposition for the SRES projection A1 for countries with an overall effect exceeding 0.01 (1%). China contributes the largest absolute amount, this is primarily driven by changes in the ratio of China's GDP relative to aggregate GDP. The largest CO_2 intensity growth rate effects stem from Russia and South Africa, however, the total effect of these is comparatively minor due to their small weight in global emission intensity.

Figure S7: Country-by-country contribution to the Global Difference between Observations and Scenario Projections (A1) in Growth Rates 2000-10 of Emission Intensity Decomposed into CO_2 intensity Growth (blue), GDP (green) and Total Effects (orange).

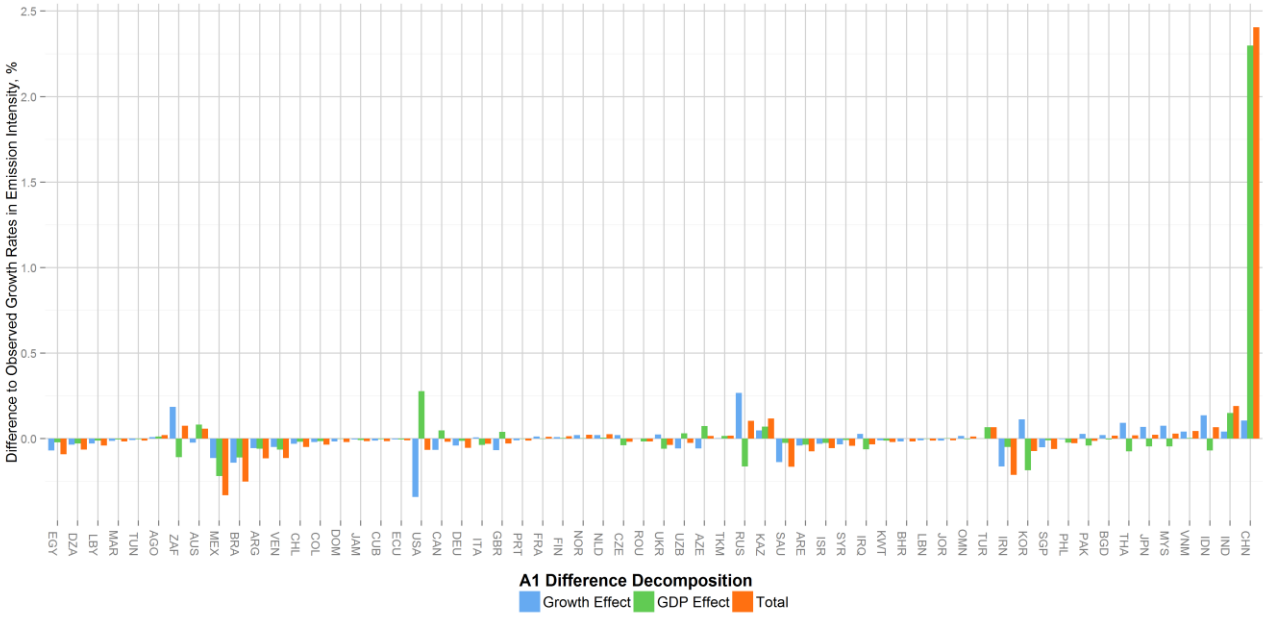

S4 Observed Country-by-Country Growth Rates of Emission Intensity

Figure S8: Observed average decadal growth rates of Emission Intensity (CO_2 per GDP) by country over 2000-2010.

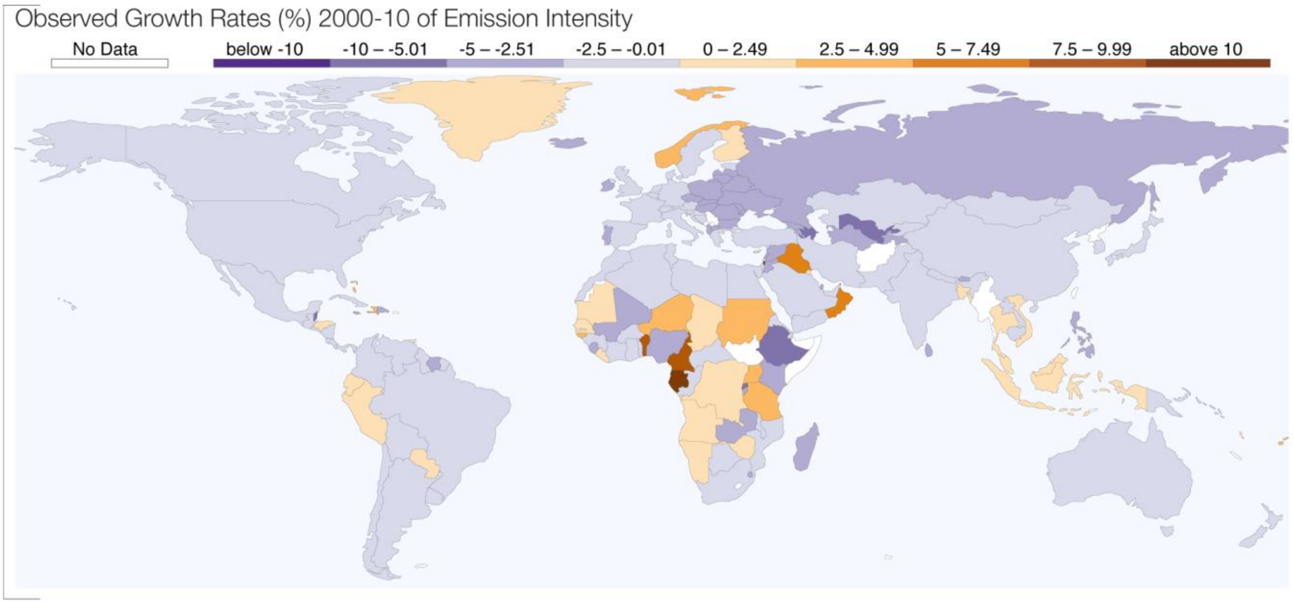

S5 Fossil-fuel CO_2 Emissions per GDP per Capita

Here we present results of emission intensity measured as fossil-fuel CO_2 emissions per GDP per capita. Consistent with the results in the main text, decadal emission intensity per capita growth over the 2000s exceeded all IS92 and SRES marker scenarios (Figure S9).

Figure S9: Observed and projected global emission intensity measured in fossil-fuel CO_2 emissions per GDP per capita in levels *(a)* and growth rates *(b)*. Panel *a* graphs global observed annual fossil-fuel CO_2 emissions per GDP (black) together with decadal IS92 (dashed colour) and SRES marker projections (solid colour). Starting values are shaded grey while projected values are shaded light blue. Note that initial values for SRES vary across scenarios. Panel *b* shows observed annual growth rates (continuous black) together with observed decadal growth rates (horizontal black) over both decades. Projected growth rates are shown in colour for IS92 (dashed) and SRES marker projections (solid). Observed decadal growth rates exceed all scenario projections over the 2000s.

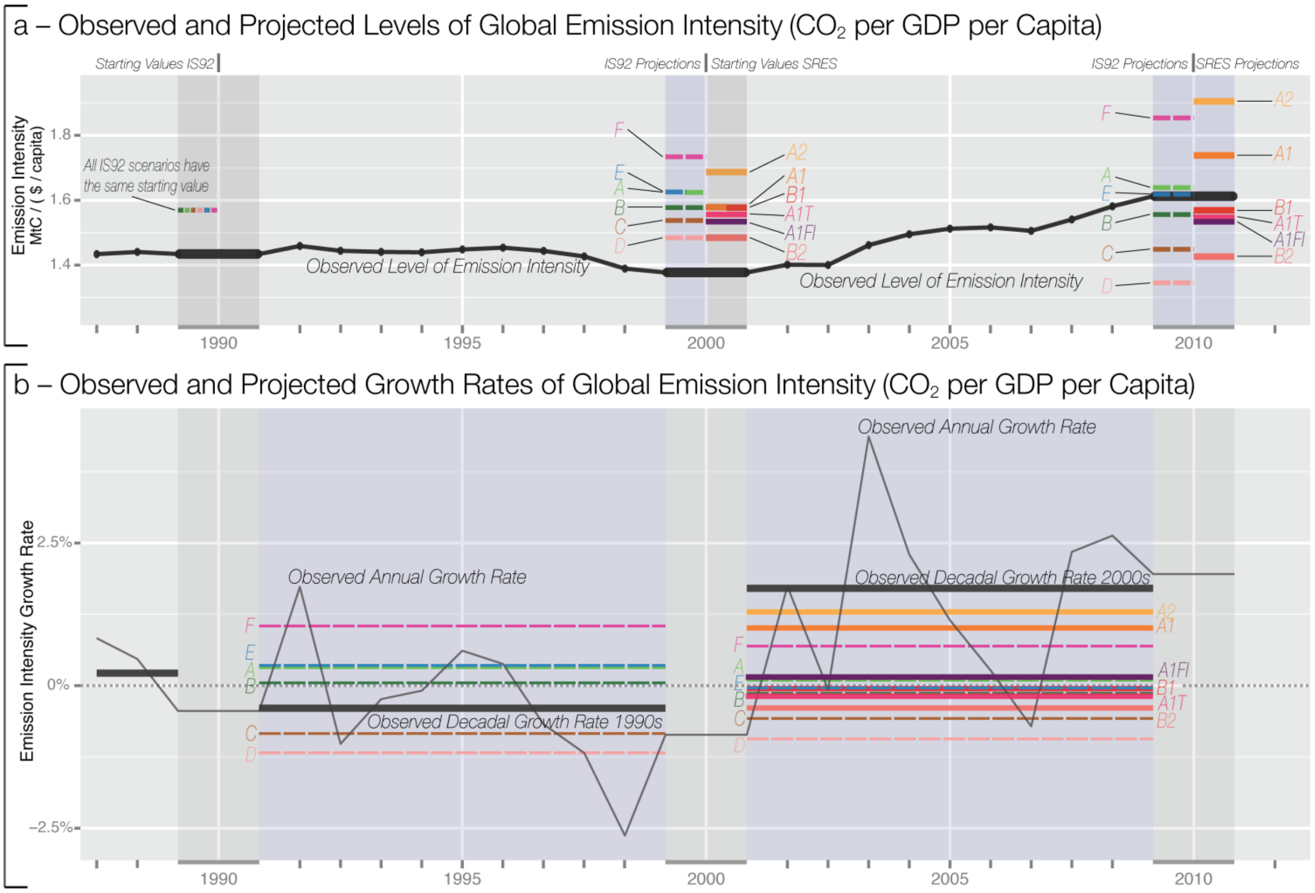

S6 Alternative down-scaling method using data from van Vuuren et al. (2007)

Figure S10: Decomposition of the global difference between global observed and projected emission intensity using the van *Vuuren et al. (2007) country-by-country downscaled difference between observed and projected decadal growth rates in emission intensity for SRES marker projections over 2000-2010.* The panel graphs the country-by-country contribution to the difference between global observed and global projected growth rates in emission intensity. Only countries contributing more than 0.001 towards the difference are shown. The primary contribution to the differences in global growth rates using relative to SRES projections stems from changes in Asia and China in particular, while some (e.g. US) observations remain close to projections, thus lowering the difference between global observed and projected growth rates.

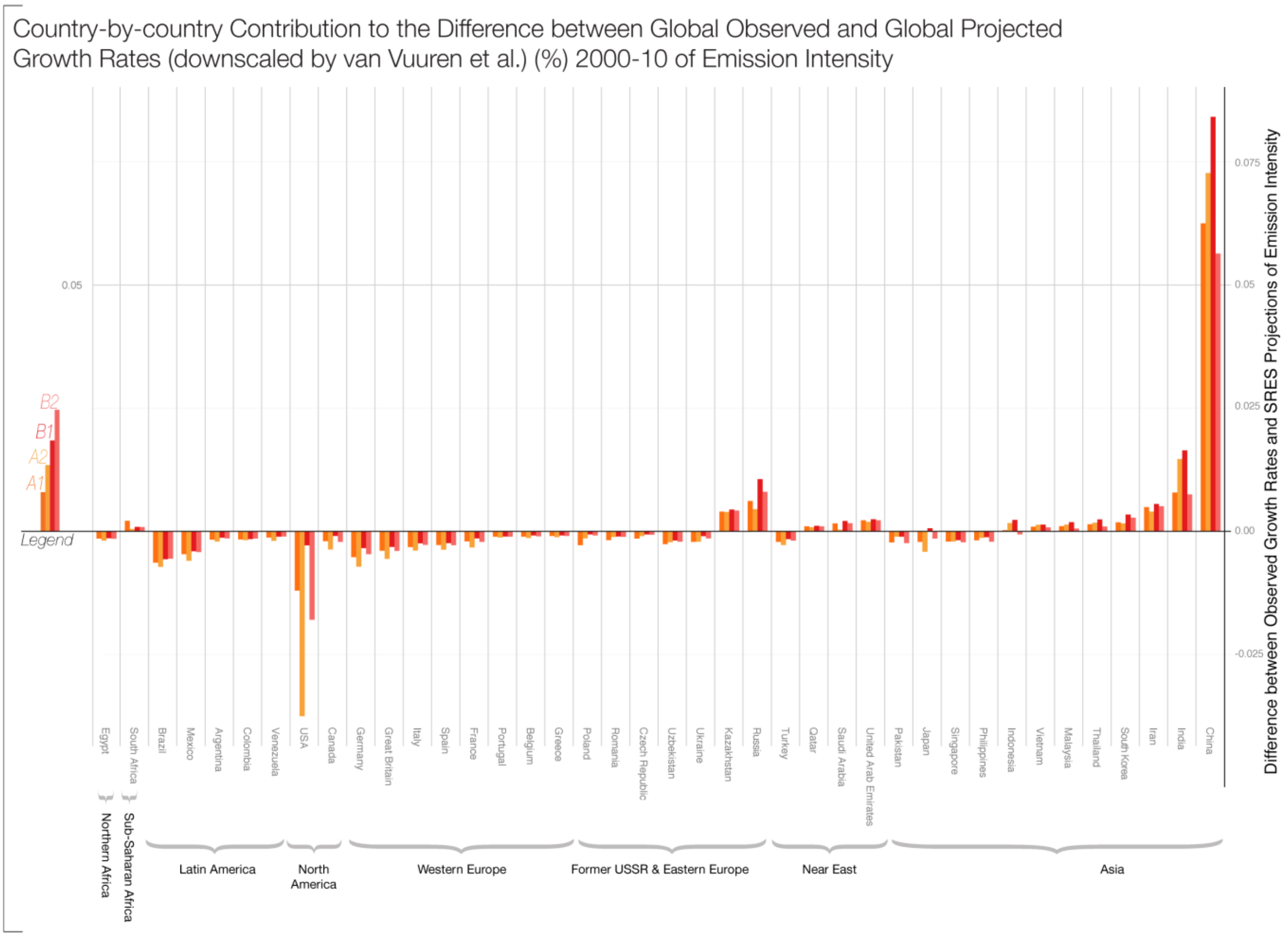

Supplement: Supplementary file 1 [file mmc1.pdf]
